# Cultivating a Science, Technology, Engineering and Mathematics (STEM) community for two-year college student success and persistence

**DOI:** 10.1371/journal.pone.0290958

**Published:** 2023-09-08

**Authors:** Deann Leoni, Tom Fleming, Jenny L. McFarland

**Affiliations:** 1 Mathematics Department, Edmonds College, Lynnwood, Washington, United States of America; 2 Physics Department, Edmonds College, Lynnwood, Washington, United States of America; 3 Biology Department, Edmonds College, Lynnwood, Washington, United States of America; University of New England, AUSTRALIA

## Abstract

Undergraduate students studying Science, Technology, Engineering and Mathematics (STEM) often fail to persist in critical “gateway” courses, resulting in students leaving the STEM pathway. Community college students leave STEM pathways at higher rates than students at universities. Implementation of a program designed to engage community college STEM students and faculty in a community of support was associated with increased persistence in STEM gateway courses and associate degree completion. Program elements included support staff, a STEM study room with peer tutors, faculty advisors, and transfer assistance. Over seven years, 415 students joined this opt-in support program. The majority of students in this program were economically disadvantaged and many were nontraditional college students. Using institutional data we tested the hypothesis that participation in this program was associated with increased student success and persistence in STEM courses and at the college. The mean GPA for students in the program in the ten courses with the highest STEM enrollments was higher (2.89) than that for other students (2.76). Quarter-to-quarter persistence was 87% for program students compared to 67% for non-program students in a matched student population. In STEM gateway courses, program students had between 1.2x to 3.5x greater likelihood than non-program students of progressing to precalculus-2 controlling for first-attempt GPA in precalculus-1. Similar persistence patterns were observed for other gateway STEM courses. Observed persistence for students in the program was higher than comparable groups of students, including persistence for those who experienced early failure in STEM courses. These data suggest students should be supported through early failure to enable persistence in critical STEM sequences, especially in gateway Math and Chemistry courses.

## Introduction

In the past decade, between 33 and 40 percent of U.S. undergraduates were enrolled at community (two-year) colleges. These institutions will continue to provide an important foundation for students earning degrees in science, technology, engineering, and mathematics (STEM) [1,2] despite declining community college enrollments [3]. Community colleges typically enroll a higher percentage of non-traditional students (older than 22) and students from historically marginalized communities than four-year institutions, and they can play a pivotal role in increasing the diversity of the STEM workplace [4]. However, the large population of students at community colleges has often been ignored in critical conversations about STEM education, workforce shortages, and economic challenges [5,6]. National discourse, research, and funding in higher education for STEM is predominantly centered on four-year institutions. As more STEM majors and a more diverse workforce are required to fulfill 21st-century challenges, more research is needed to understand this important portion of the undergraduate population [7,8].

An important role of public community colleges is to provide equitable access to higher education [9]. Community colleges provide open access to higher education regardless of secondary education grades or test scores. Two-year colleges enroll students who are economically disadvantaged, attended high schools with limited STEM resources [10], and are more likely to be historically excluded in STEM [11]. Community colleges’ relatively low tuition and fees and regional locations allow students to live at home and continue to work [12] and thus provide an important foundation and pathway for students who want to earn STEM degrees [6,13–15].

### CC student persistence

Although there is substantial student interest in STEM careers, U.S. college students continue to leave STEM fields at high rates. Fewer than half of first-year undergraduate students who start at a college or university in a STEM field graduate with a bachelor’s degree in STEM six years later [16]. Attrition rates of community college students tend to be substantially higher than students at four-year universities [17,18]. Although there are many potential reasons for these lower persistence rates, research has shown that implementation of high-impact practices and a range of opportunities focused on STEM student success may increase student retention and academic success of historically marginalized populations [19] and influence community college transfers’ STEM degree attainment [20,21].

Introductory science and mathematics ‘gateway’ courses, including general chemistry and calculus, prepare students for future STEM courses but can also act as barriers to persistence in STEM pathways [13,22,23]. STEM-interested college students are often ‘weeded out’ or lose academic momentum as they attempt to complete prerequisite gateway courses in chemistry [24] and mathematics [25], and disadvantaged or underrepresented students are more likely to be impacted by these barriers. Loss of academic momentum, the rate at which students earn credits particularly in their initial terms, may decrease the probability of completing an undergraduate degree and working in a STEM field in the future [26–28]. Providing academic support in gateway STEM courses has been shown to increase persistence [29]. For example, the University of Maryland-Baltimore County Meyerhoff Scholars Program has had success increasing their number of STEM undergraduates through a program that includes tutoring, peer support, personal advising, and other elements designed to reduce student isolation and barriers that result from unsupportive learning environments [30]. Community colleges have opportunities to support STEM students and increase persistence through their critical introductory courses. The mission of community colleges is focused on undergraduate education and students have more access to faculty through smaller class sizes, faculty-taught laboratories, and ample office hours.

### The RiSE program

In this study, we evaluate STEM student success and persistence over seven years of the Relationships in Science Education (RiSE) project at Edmonds College (formerly Edmonds Community College), a public two-year college in the Western United States. The RiSE program was a student-centered, faculty-driven program, designed to increase the number of students earning STEM associate degrees and transferring to a university to complete a STEM degree. The RiSE program was designed using knowledge that relationships cultivated within a specific support program, particularly student-faculty relationships, may help students academically and socially [31–34]. The program name, *Relationships in Science Education*, exemplifies the key focus of the program: to create relationships that provide academic and personal support for STEM students. Advising, mentoring, enrichment events, and student activities provided opportunities for relationships to build among STEM students, faculty, and staff outside of the classroom. Many elements of this program were developed based on evidence-based practices, including advising and supplemental instruction, which have been shown to positively affect retention of students [35–37]. A primary underlying assumption was that external, environmental barriers could be addressed by building community and increasing access to resources, instead of ‘fixing student deficits’ [38,39].

The RiSE program consisted of academic and personal support elements and social, career, research, and service activities including those in Table 1.

**Table 1:** RiSE program elements and associated intended benefits.

Program Element	Intended Benefits
STEM study room	Academic support through tutors; community with other STEM students
Undergraduate Research experiences	Increased identity as a scientist; lab and research experience
Field trips (enrichment, industry, and university-transfer)	Exposure and experiences
Student events (social, academic, transfer)	Building a community
Service learning opportunities	Increased identity as a scientist
Advising/mentoring by faculty members in their field and STEM support staff	Personal support and transfer assistance
Peer-tutors and supplemental instruction	Academic support

### Research questions: Student persistence and success

In order to evaluate the RiSE program, we asked the following research questions:

1. Was participation in the RiSE program associated with student persistence?
2. Was participation in the RiSE program associated with student academic success? ​

Persistence at the college and in particular STEM course sequences were used as measures of student persistence. See Appendix A (Table A1 in S1 File) for specific definitions and examples of persistence used in this paper. Measures of student success were overall GPA, grades in specific STEM courses, and completion of a transfer degree.

## Methods

The use of institutional demographic, enrollment, grade, and degree data were deemed exempt by the Edmonds College Institutional Review Board. This work was performed using practices that are not likely to adversely impact students’ opportunity to learn and met the category 1 exemption criteria.

### RiSE student population

We describe the students’ age range and enrollment patterns to illustrate the complexities of two-year college students. The data in the paper includes the RiSE cohort of 362 students who joined RiSE between the start of the project in Fall 2011 and Spring of 2017.

#### Student age

**Fig 1:**
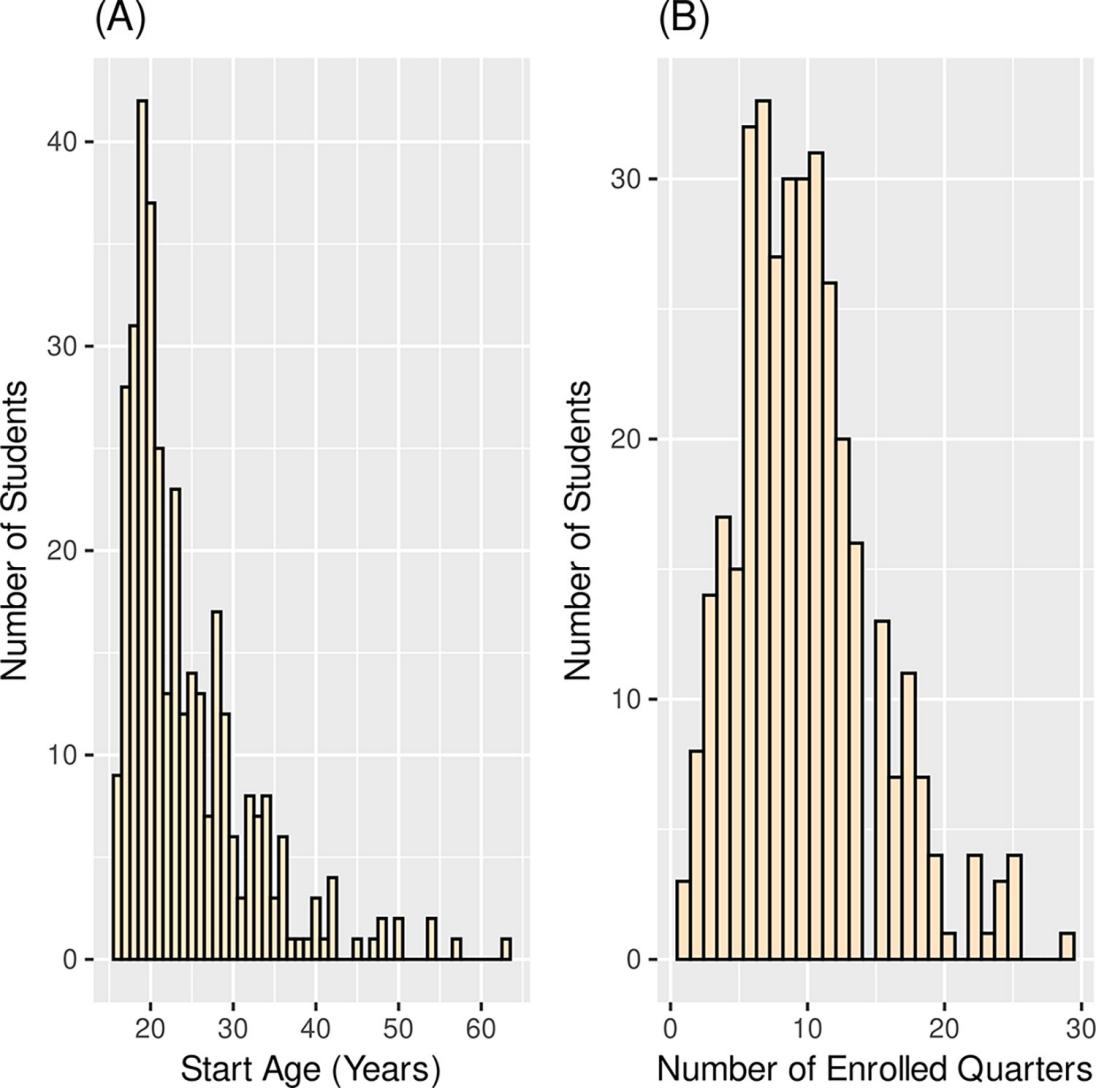
(A) Age distributions of RiSE students at the time they joined RiSE. Seventeen of the 362 RiSE students are not included here due to undeclared dates of birth. (B) Distribution of the number of enrolled quarters of RiSE students through Spring quarter 2017.

RiSE students ranged in age from 16 to 63 years of age and the mean age of RiSE students at the time they joined rise is 24.2 years. Like other community college student populations, RiSE students, on average, were older than traditional college students, and Fig 1A demonstrates that this population is not normally distributed. Students who joined RiSE in the period Fall 2011 through Spring 2017 were actively enrolled at the college for anywhere between one and 29 quarters, with a mean of 10 quarters, and middle-50 percentile of RiSE students having been enrolled between 7 and 13 quarters. (Fig 1B).

#### Financial disadvantage

**Fig 2:**
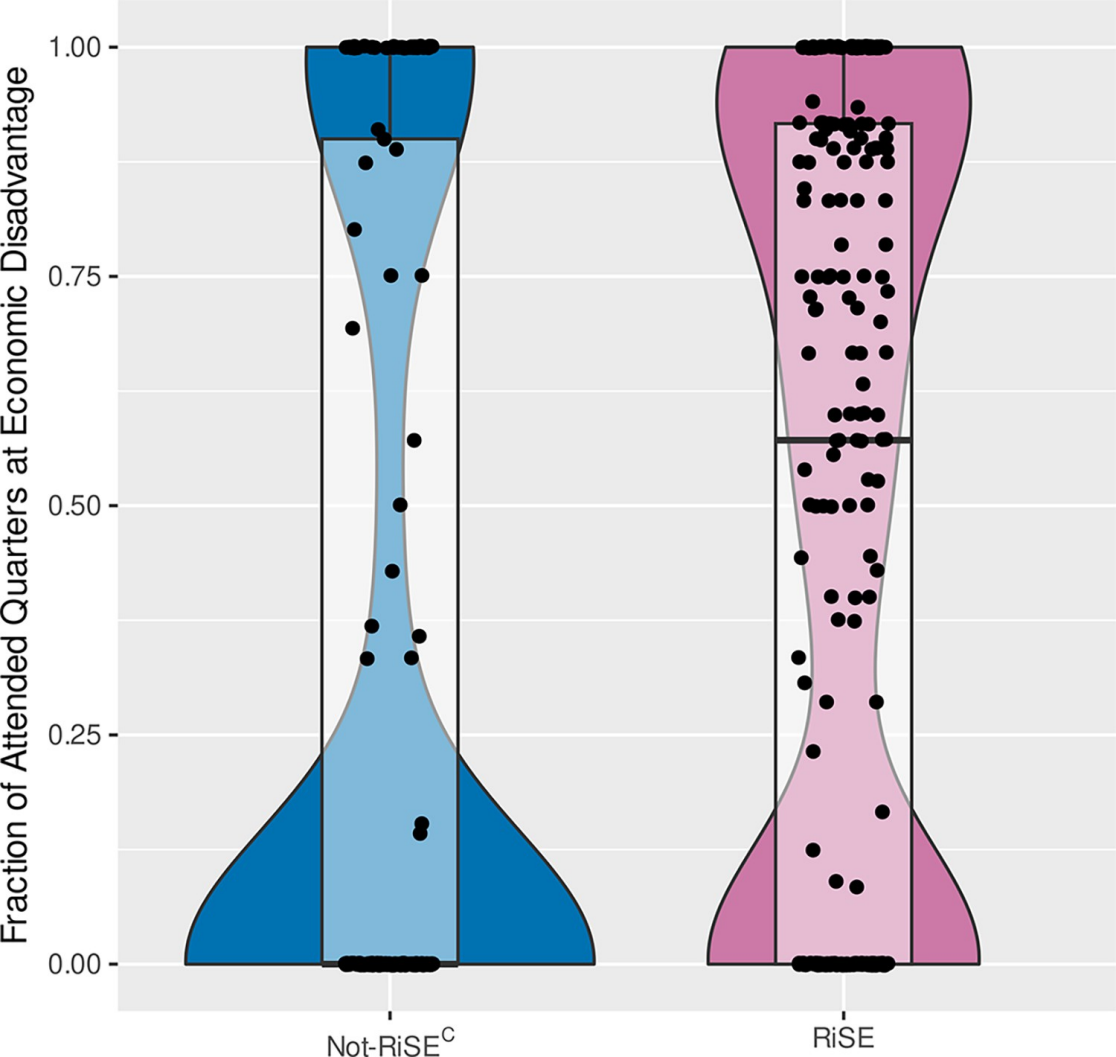
Distribution of the fraction of enrolled academic terms (quarters) that a student was at economic disadvantage for students in the RiSE and the Not-RiSE^C^ comparable populations. The transparent boxplots show the quantile distributions, with a RiSE student population median of 57.1% of attended quarters at economic disadvantage compared to a median of 0.0% for the comparable, Not-RiSE^C^ population. The horizontal widths of the violins are densities, with the total area of each violin normalized to 1.

As is common at two-year colleges, the RiSE students are predominantly economically disadvantaged, with 222 (62%) having qualified as economically disadvantaged (defined as receiving need-based financial aid, including need-based waivers) at least once during their quarters of enrollment (see Fig 2). Furthermore, the intensity of economic disadvantage among RiSE students is noteworthy in that more than 50% of RiSE students qualified for financial aid in more than 70% of the quarters they were enrolled at the college. Fig 2 summarizes the per-student distribution of the fractional number of enrolled quarters that RiSE students who qualified as economically disadvantaged for at least one quarter spent in a condition of economic disadvantage. In this figure, the RiSE population is compared to Not-RiSE^C^, one of the comparison populations described below and summarized in Appendix B (Table B1 in S1 File).

Seventy-one of the 362 RISE students (19.6%) received a scholarship for low-income STEM students for at least one quarter between Winter 2012 and Winter 2017. Although separate from RiSE, the scholarship program worked collaboratively with RiSE, and all of the scholarship recipients were required to join RiSE and were encouraged to participate in RiSE events and seek additional academic and personal support through RiSE.

### *Research question 1*: Measures of student persistence

#### Quarter-to-quarter persistence

**Fig 3:**
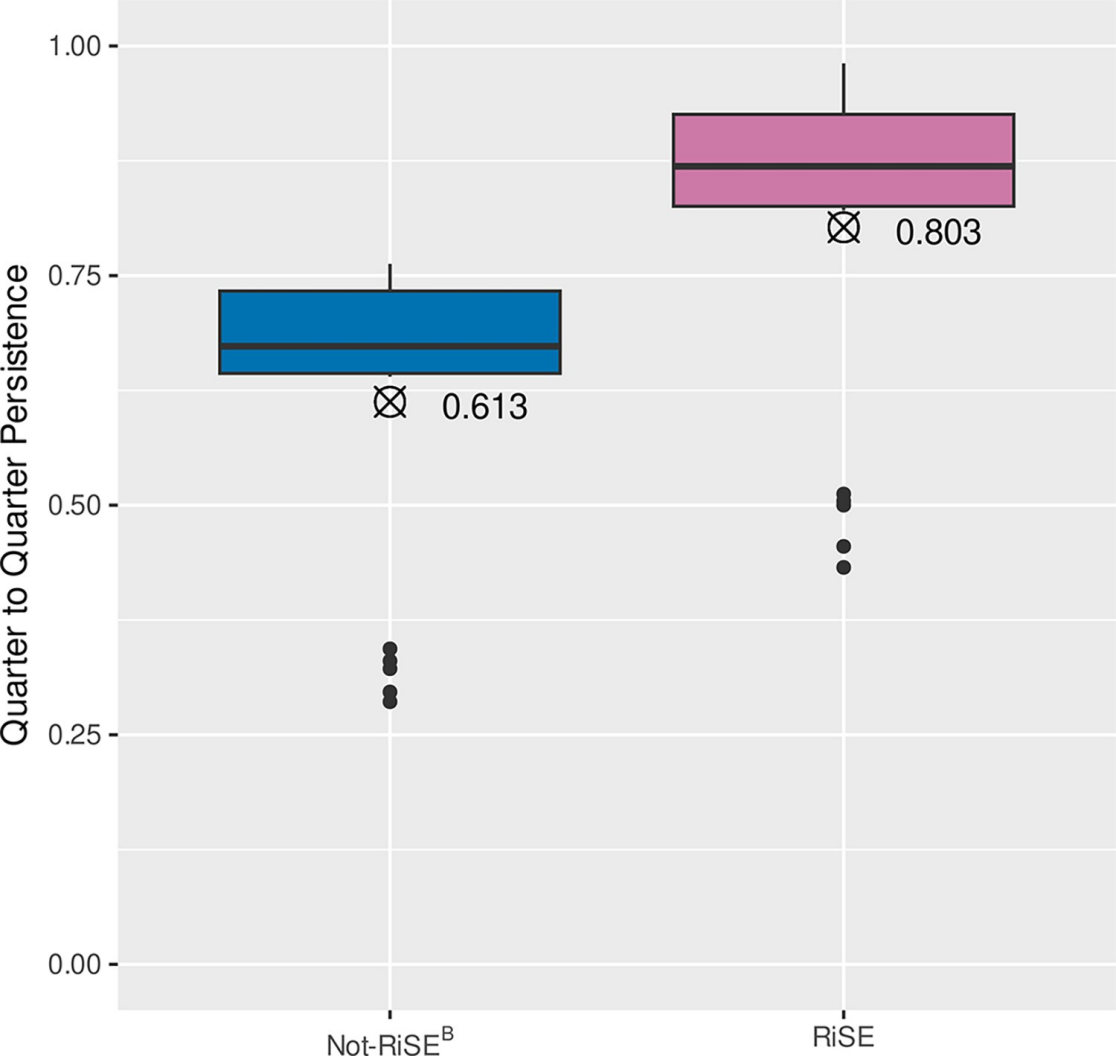
Over N_Q_ = 22 consecutive quarters, the median persistence rates for RiSE students (0.87) are significantly higher than for Not-RiSE^B^ students (0.67), but in the same classes. The outliers correspond to Summer quarter persistence, which is typically lower as many students choose to not enroll over Summer quarter.

Quarter-to-quarter persistence refers to the fraction of students in a specific academic term who were enrolled in the following academic term (excluding those who completed degrees or certificates) (see Table A1 in S1 File). Enrollment data was obtained and analyzed over 23 quarters from Fall 2011 to Spring 2017, including summer quarters, providing 22 pairs of consecutive quarters. These data (Fig 3) included 314 RiSE students and 1061 Not-RiSE^B^ students who were in the same sections of the same courses in the same term (including, but not limited to, STEM courses) over these quarters.

#### Persistence in gateway STEM course sequences

Persistence in gateway STEM courses refers to a student’s probability of enrolling in the next course in a sequence, given that student’s grade in their first attempt at the preceding course in the sequence. Students in non-STEM “general education” classes may differ in motivation, goals, and preparation compared to students in courses intended for STEM transfer degrees. Thus, a better comparison group was needed to examine persistence of RiSE students more accurately in STEM gateway sequences and completion of transfer degrees. To compare the persistence of students in RiSE with similar students who were not in the program we identified a Not-RiSE comparable population, Not-RiSE^C^, as summarized in Appendix B (in Table B1 in S1 File) and whose formation is described in detail in Appendix C.

The Not-RiSE^C^ comparable population was formed by drawing a random sample of N = 10,000 Not-RiSE^C^ students with enrollments over the period of first known RiSE student enrollment (Fall 1995) through the Spring 2017. Any students who had not taken both at least one Math and one English course at the college were eliminated from the comparable population because we were looking for an indicator of preparedness for college-level courses. Using these populations and an initial candidate set of 18 student-records database indicators, we used the R glmulti package [40] to conduct an exhaustive search of binary logistic regression models (without interaction terms) minimizing AIC_c_ in order to find the most parsimonious classification model capable of distinguishing between RiSE and Not-RiSE^C^ students. The minimum-AIC_c_ model retained 11 of the original 18 indicators. The AIC_c_-minimized glm model on the 11 retained indicators was then used as the propensity distance measure [41] of RiSE membership along with a genetic algorithm method as implemented by the R package MatchIt [42], which calls functions from the R Matching package [43,44] to obtain matched RiSE and Not-Rise^C^ populations balanced on the on the 11 retained indicators. After balancing on the 11 retained indicators, the final populations retained for comparison consisted of N(Not-RiSE^C^) = 6634 and N(RiSE) = 299 students. Five of the RiSE students were discarded because no Not-RiSE^C^ matches could be found for them (they did not have sufficient propensity overlap with anybody in the Not-RiSE^C^ population), and 5745 Not-RiSE^C^ students were discarded from the candidates for the comparable population. The formation of the comparable population, the indicators used, and regression coefficients for the propensity model are described in more detail in Appendix C (Table C1 and Fig C1 in S1 File).

We examined persistence in math (courses leading to calculus) and chemistry (general chemistry), which are sequences required for most STEM majors. We used a logistic regression model to compute the likelihood that a student would ‘arrive’ (i.e., enroll) in a particular class *C*_*n*_ in a sequence based on their grade *G*_*m*_ in the preceding course *C*_*m*_ and whether they were in the RiSE program or in the comparable population (Not-RiSE^C^).

We assume a simple functional relationship between the log-odds of "arrival" in course *C_n_* of the form $z(A_{n}|G_{m},R)=\ln(\frac{p(A_{n}|G_{m},R)}{1-p(A_{n}|G_{m},R)})$

$$=\beta_{0}+\beta_{m}G_{m}+\beta_{R}R$$

where $p(A_{n}|G_{m},R)$ is the probability of arrival in course *C_n_*, given grade *G*_*m*_ received in preceding course *C_m_* and RiSE membership, *R*.

This model is explained in detail in Appendix D of S1 File. We used this model to define the comparative advantage effect size into an effective course grade advantage by comparing the differential increase (or decrease) in grade points required by Not-RiSE^C^ students in a particular course, *C*_*m*_, to have the same persistence probability of RISE students. Thus, for any fixed grade, *G*_*m*_, we could compute a predicted differential increase (or decrease) in grade by Not-RiSE^C^ students required to make the comparative persistence advantage of Not-RiSE^C^ students equal to that of RISE students.

### *Research question 2*: Student success

Course grades, GPA (grade point average) and completion of transfer degrees are all measures of academic success [45]. To analyze the comparative measures of “success” we looked at two different comparisons, comparing GPAs of students in the most enrolled STEM courses and time-to-completion of first transfer degree.

#### GPA in top-10 STEM courses

As a measure of academic “success” we wanted to know how the mean GPA of RiSE students compared to that of Not-RiSE^A^ students in STEM courses (see Table B1 in S1 File for a description of the Not-RiSE^A^ comparison population). We looked at student grades in the ten STEM courses with the highest annual enrollments to determine the “top-10 STEM courses”. These high-enrolled courses included three general Chemistry courses (CHEM& 139, 161 and 162), two Physics courses (PHYS& 221 and 222) and five Mathematics courses (Precalculus 1 & 2: MATH& 141 and 142 and Calculus 1–3: MATH& 151, 152 and 153). A direct comparison of student grades is difficult because the answer depends simultaneously on (1) the course, (2) the quarter, (3) the number of RiSE students in the course and (4) the number of Not-RiSE^A^ students in the course.

Differences between RiSE and Not-RiSE^A^ mean class GPAs for students within the same top-10 STEM courses were compared using an N = 100,000 bootstrapping resampling of RiSE and Not-RiSE^A^ student GPAs by course, year and quarter [46]. Of the N = 100,000 samples, 1124 non-comparable samples (due to course sections with either no RiSE students or no Not-RiSE^A^ students registered) were dropped leaving N(RiSE) = N(Not-RiSE^A^) = 98876 top-10 STEM course mean GPA samples for each group. We compared these resampled mean GPAs by group using the Wilcoxon-Mann-Whitney two sample rank-sum test with rank-biserial (r) correlation as an estimator of RiSE effect size [47].

The sampling of mean GPAs from randomly selected courses allows for the GPAs to contribute to the total according to their natural weight, i.e., GPAs are weighted according to the underlying probability of the students taking a particular course, which is a priori unknown. GPAs from low-offering and low-attendance courses are weighted less than high-offering and high-attendance courses. In this way, the calculation is more representative of actual student course-taking behavior (see Appendix E in S1 File).

#### Time-to-completion of transfer degree

We compared the time to degree completion for RiSE and Not-RiSE^C^ populations (see Table B1 in S1 File). A Kaplan-Meier survival analysis of time-to-event was performed, where time is the number of quarters of active enrollment at the college, and the event of interest is observation of a student completing a transfer degree as their first-attained award. The observational options available were either: (1) the student is observed to have an event (attain a first transfer degree during the observation period), or (2) to censor during the observation period (to be lost to follow-up, either by permanently electing to not return to complete, to go elsewhere to complete, or simply not having enough time to complete during the time period of the study). As long as a student continued to enroll without observation of an event (transfer-degree completion), they were still "at risk" of completion. Students who stopped attending were no longer “at risk” of completion.

### Student intent

Student intent data was obtained from registration forms that included “Student Intent Codes,” including Job Skills, Transfer, Developmental Education, and other options. Identifying STEM transfer students is not possible without declared majors at the college, so these codes are used instead. Although many students do not change “intent” during the period they are enrolled at the college, students can and some do change, and these data were obtained for the RiSE students.

## Results

### *Research question 1*: Student persistence

#### Quarter-to-quarter persistence

We first examined persistence at the college, whether students ‘returned’ and enrolled in successive quarters. Over the N_Q_ = 22 consecutive quarters studied, the median quarter-to-quarter persistence rates were roughly 30% greater for RiSE students (Mdn = 0.87, n_RiSE_ = 314) than for Not-RiSE^B^ students (Mdn = 0.67, n_NotRiSE_ = 1061). Comparing the quarter-to-quarter persistence rates of the two populations using the Wilcoxon-Mann-Whitney rank sum test, we take as an effect size the Hodges-Lehmann estimator (HLΔ) of the median of all (N_Q_ x N_Q_) = 484 pairwise differences between RiSE and Not-RiSE student quarter-to-quarter persistence rates, yielding a median increase in RiSE student quarter-to-quarter persistence relative to Not-RiSE students of HLΔ = 0.192 (95CI[0.149, 0.244], W = 399, p = 0.00024, two-tailed). See Fig 3.

### Persistence in gateway STEM course sequences

We compared RiSE student persistence in STEM gateway course sequences to students not in the RiSE program (Not-RiSE^C^). In this analysis of persistence, we used a propensity score model that resulted in a population of 260 Not-RiSE^C^ students to compare with 294 matched RiSE students. Persistence here is the ratio of RiSE vs. Not-RiSE^C^ student persistence probability, given the student’s grade in their first attempt at the preceding course in the sequence.

**Fig 4:**
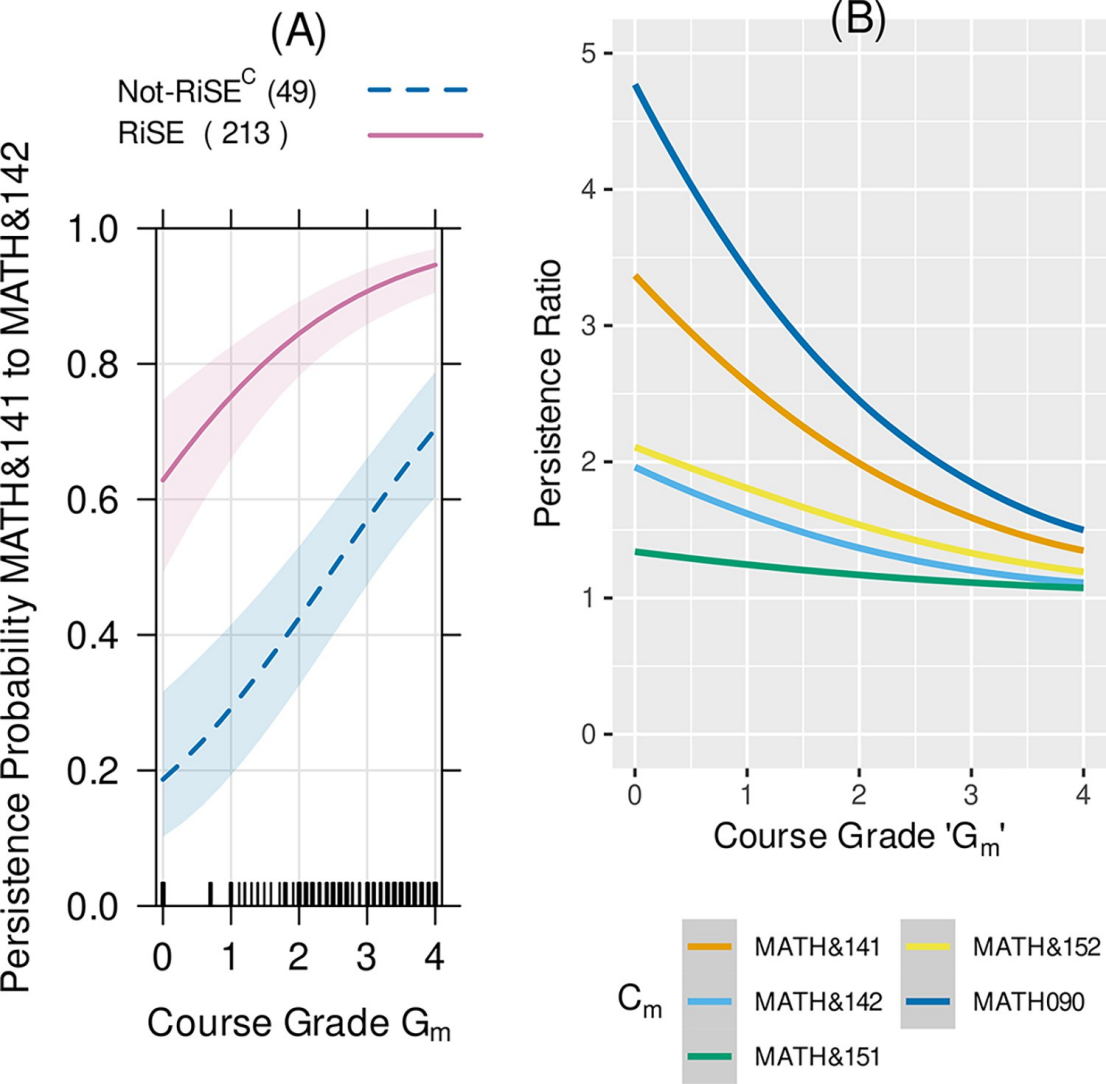
(A) The probability of persistence for RISE students (solid line) and Not-RiSE^C^ students (dashed line) in precalculus. The shaded regions show the 95% confidence intervals for the model fits and the "rug" plots on the horizontal-axis show the relative numbers of students at each grade level in each course-pair-persistence-sample. This shows the probability of enrollment in Precalculus 2 (MATH& 142) vs. the student grade in their first attempt in Precalculus 1 (MATH& 141). (B) The persistence probability ratio for (A) is the second from the top line in (B). The persistence probabilities ratios show that RiSE students were more likely to continue from one math course to the next and this effect was largest for Intermediate Algebra to Precalculus 1 and from Precalculus 1 to Precalculus 2.

Here we report the logistic regression analysis of the data for the Math and General Chemistry sequences, as those courses are required by all STEM degree pathways. RiSE students had between 1.2x to 3.5x greater likelihood than Not-RiSE^C^ students of progressing from Precalculus 1 (Math& 141) to Precalculus 2 (Math& 142) at every first-attempt Precalculus 1 grade level (Fig 4A). The shaded areas in Fig 4A show the 95% confidence intervals of the predictions from the logistic regression model. The parameter estimates and their confidence intervals are given in Table D1 in S1 File. For example, RiSE students with a grade of a 2.0 (C grade) in their first-attempt in Precalculus 1 had a persistence probability of 0.85, whereas Not-RiSE^C^ students with grade of 2.0 had a persistence probabilities of 0.4, and Not-RiSE^C^ students with a grade of 4.0 (A grade) had a probability of 0.7. The number of RiSE students in the matched sample who progressed from Precalculus 1 (MATH& 141) to Precalculus 2 (MATH& 142) is 1.78 times the number of Not-RiSE^C^ students in the matched sample who progressed from Precalculus 1 (MATH& 141) to Precalculus 2 (MATH& 142) (e.g., second line from the top line in Fig 4B). Another example using the parameters to calculate course persistence is shown in Appendix D in S1 File.

**Fig 5:**
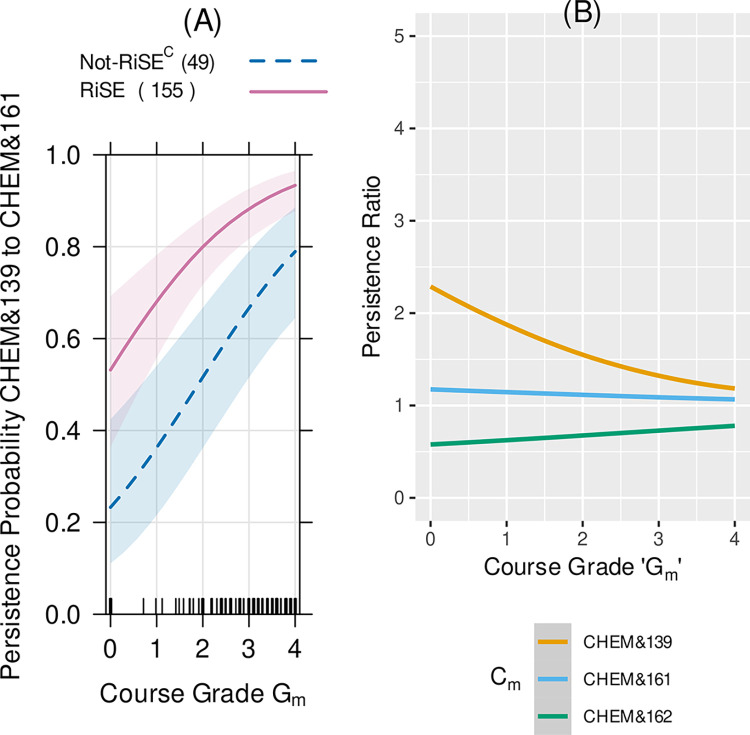
(A) The probability of persistence, that is enrollment in General Chemistry 1 (CHEM& 161) vs. the student grade in their first attempt in the Chem Prep course (CHEM& 139) for RISE students (solid line) vs. Not-RISE^C^ (dashed line). The shaded regions show the 95% confidence intervals for the model fits and the "rug" plots on the horizontal-axis show the relative numbers of students at each grade level in each course-pair-persistence-sample. (B) The persistence probability ratio for (A) is the top line in (B). The persistence probabilities ratios show that RiSE students were more likely to continue from the Chem Prep course to general chemistry.

Similar persistence patterns were observed for other gateway STEM courses, including biology, chemistry and calculus sequences. For example, in Fig 5A, the probability of a RiSE student who earns a grade of 1.0 (D grade) in Chemistry Prep (CHEM& 139) has the same probability of persistence in the Chemistry sequence, that is eventually enrolling in General Chemistry 1 (CHEM& 161), as a Not-RiSE^C^ student in the comparison population with a 3.2 (B grade) in the Chemistry Prep course.

### *Research question 2*: Student success

#### GPA in top-10 STEM courses

**Fig 6:**
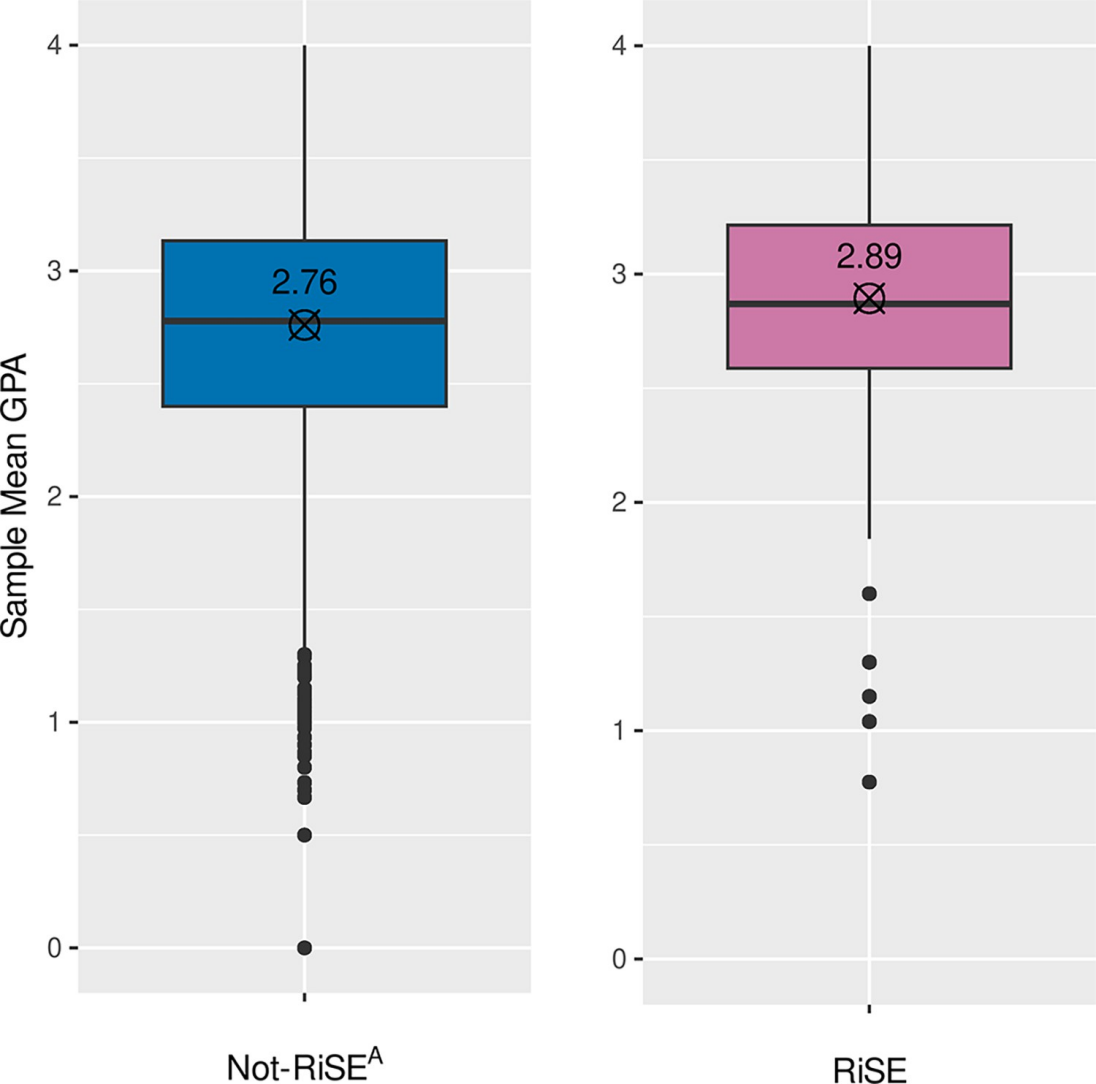
Mean GPA of students in RiSE in the top-10 STEM Courses, Fall 2011 through Spring 2017, compared to students in the same course sections but not in the RiSE program, Not-RiSE^A^. The numbers and crossed circles mark the means of the mean GPA distributions.

The GPAs of RiSE students in STEM courses were compared to those of students who were not in the RiSE program (Not-RiSE^A^). The mean GPA for students in the top-10 STEM courses at the college from September 2011 to June 2017 for RiSE students was higher (2.89) than that for other STEM students in the same classes who were not in the RiSE program (2.76), as shown in Fig 6. Using the Wilcoxon-Mann-Whitney two sample rank-sum test, the mean top-10 STEM course GPAs of RiSE students was greater than the mean GPAs of the Not-RiSE^A^ students in 56.4% of the samples, defined as a small effect [47] (r = 0.128, 95CI[0.123,0.133], W = 5512050174, N(RiSE) = N(Not-RiSE^A^) = 98876, p = 0.000, two-tailed).

Some RiSE students also received STEM scholarships, and the same analysis was conducted for this subset of RiSE students who received a scholarship for low-income STEM students, and the mean GPA in the top-10 STEM courses for the scholarship students was 2.90.

### Time-to-completion of transfer degree

**Fig 7:**
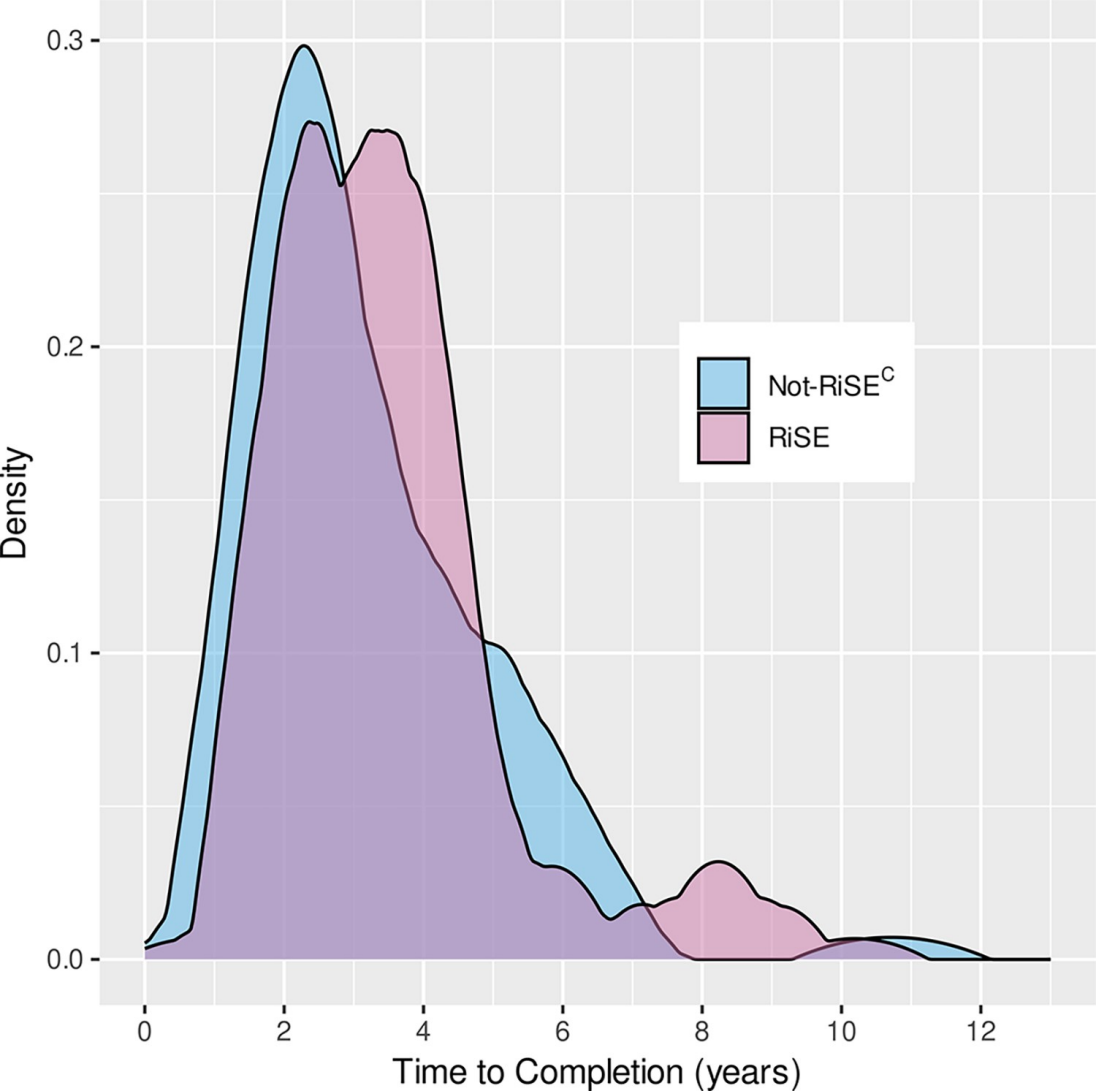
Time-to-completion of first awarded transfer degree. Delta plot (Epanechnikov kernel smoothing) of the probability density of the “risk” of completing a transfer degree for the population of RiSE students and the population of Not- RiSE^C^ STEM students.

During the time period of this study, 34% of RiSE students completed an associate degree, compared to 27% of Not-RiSE^C^ students (Fig 7). For the Not-RiSE^C^ population there was a single, prominent peak in the distribution at just over 2 years. However, the RiSE population distribution had two high peaks, with a second peak occurring near 3.5 years.

### Student intent

**Fig 8:**
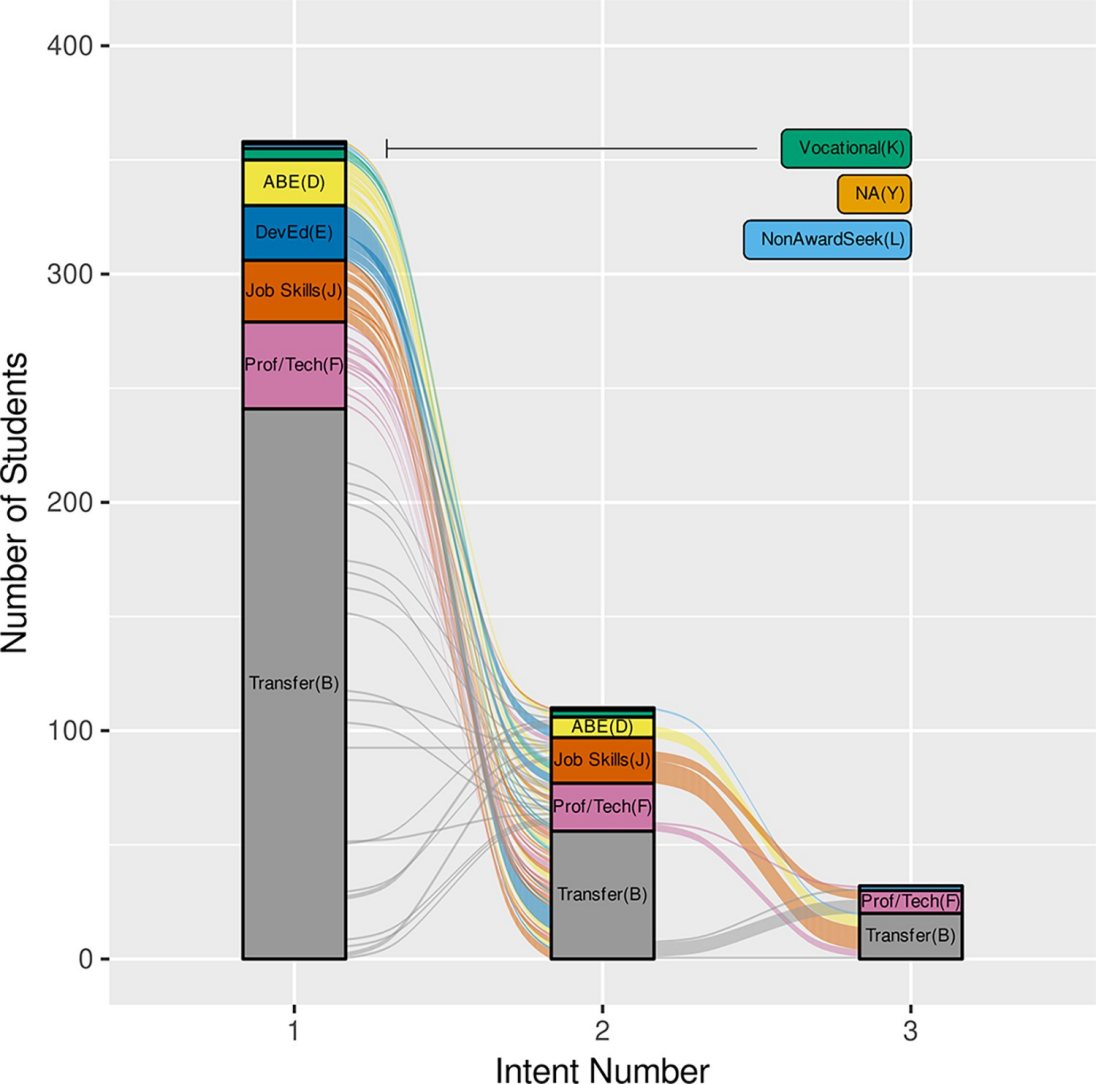
RiSE student intent transitions from initial intent during first enrollment at the college to second intent (n = 110) and then to a third intent for some (n = 31). The 252 RiSE students who did not change their original intent are not shown for clarity.

Although it seems reasonable to assume that a STEM-identifying student would enroll with an intent to transfer, Fig 8 shows that only 20% of RiSE students indicated an intent to transfer at the time of their first enrollment. Larger proportions of RiSE students first enrolled at the college with intent to either complete developmental education courses (DevEd, 22%) or complete or enhance job skills (Job Skills, 21%), followed by adult basic education (ABE, 18%) and professional/technical certificate programs (Prof/Tech, 12%). Many RiSE students changed their intents at least once over the course of their enrollment, some more than once, and the net result was that 51% of students’ second intents flowed strongly into academic transfer. The majority of these flows into transfer intent came from students who had first enrolled at the college for developmental education or job skills purposes.

## Discussion

This paper highlights the success of a STEM student support program, implemented at a two-year college, which was associated with increases in GPA, persistence, and degree-attainment rates. Our data support our conclusions that the participation in the RiSE program was associated with: (1) STEM student persistence in gateway course sequences and at the college in general, and (2) STEM student success (GPA and transfer degree completion). Moreover, it was found that the pipeline into STEM transfer degrees increased rather than decreased as students progressed through their studies.

***Research Question 1*:** Was participation in the RiSE program associated with student persistence?

### Quarter-to-quarter persistence

There was a significant increase (HLΔ = 0.19) in the quarter-to-quarter persistence of RiSE students compared to other students at the college. This comparison included all types of courses students took at the college: general education courses and required courses. The prominent difference in these general persistence data led us to compare the persistence of RiSE students with more similar non-RiSE students in STEM courses, as we assumed that students in STEM classes may have differences in persistence compared to those in “general education” classes.

### Persistence in gateway STEM courses

Analysis of persistence from one STEM course in a sequence to the next revealed that the RiSE students had a higher probability of persistence from course to course in precalculus and calculus, biology (for life science majors), general chemistry, organic chemistry, and physics. A significant “RiSE advantage” was seen in course-to-course persistence in early STEM gateway courses, particularly in math and chemistry sequences. The persistence probability ratios (Figs 4B and 5B) show a general trend of a decrease in the “RiSE advantage” as students progressed through the mathematics courses. For example, RiSE and Not-RiSE^C^ students had a more similar probability of persistence continuing from Calculus 1 to Calculus 2 compared to the large difference going from Precalculus 1 to Precalculus 2. This decrease in a “RiSE advantage” may be explained by the change in student population and by the overall decrease in the DFW (non-passing grades of D or F or Withdrawal) [48] rate in the gateway math sequences (see Appendix F in S1 File). Specifically, the population taking the Precalculus and Calculus sequences is different because not all STEM degrees require Calculus I and II whereas all STEM degrees require completion of Precalculus 1 and 2. This may explain some of the decrease in a “RiSE advantage” in the persistence ratio for those data in Fig 4B. Similarly, RiSE students showed an increased persistence probability from the chemistry prep course (CHEM 139) to the first quarter of general chemistry, but RiSE students did not have any advantage in persistence as they progressed further along in the general chemistry sequence. This may be due to the notable decrease in DFW rates in general chemistry. The average DFW rate for CHEM 139 over the course of this study was 31% at Edmonds College (see Table F1 in S1 File). The DFW rates for the first two quarters of general chemistry were considerably lower, at 19% and 16%, respectively, over the same time period. These rates were lower than the mean DFW rates for active learning STEM courses as reported by Freeman and colleagues [49]. In these later chemistry courses, the high overall student success (low DFW percentage) may effectively eliminate any differential advantage to RiSE students.

Low performance (i.e., grades/GPA) in early college Chemistry and Math courses have been shown to be associated with students leaving STEM pathways [50,51]. Even enrolling in remedial math courses was associated with a higher likelihood of changing to non-STEM majors [52]. However, the RiSE data shows that RiSE students taking precollege math (Math 90, Intermediate Algebra, in Fig 4B) had a much higher probability of persistence than their not-RiSE^C^ classmates.

The persistence observed in RiSE students was often despite early failure. As seen in Fig 1B, RiSE students took an average of over three years to complete an associate degree. Fig 7 shows that RiSE students took longer to get this first degree compared with not-RiSE^C^ students. Yet, they persisted. The RiSE program was designed to promote a STEM community with student support from faculty, staff, and peers. Relationships with and support from others in the EC STEM community may explain, in part, why more RiSE students persisted past initial failure in gateway STEM courses.

Our data also shows that RiSE students were more financially disadvantaged than not-RiSE^C^ students (Fig 2); however, despite that disadvantage they persisted at higher rates. Community college biology students self-reported that they work for pay and have family obligations at the rate twice that of research-university students [12]. They also reported less participation in clubs and with student groups. Despite their work and family obligations, the students who opted to join RiSE engaged in a variety of STEM activities and relationship-building events.

***Research Question 2*:** Was participation in the RiSE program associated with student academic success?

### GPA in top-10 STEM courses

Fig 6 shows that the mean RiSE student GPA in the highest-enrolled (top-10) STEM courses was higher compared to the other students in those same courses (not-RiSE^A^). This finding is similar to that of the PEERS program where students in the support program had higher cumulative GPAs and higher grades in gateway STEM classes [29]. The RiSE students who received a STEM scholarship had very similar GPAs (2.90) compared to the RiSE population as a whole (2.89), so this difference in RiSE student GPA was not merely associated with receiving this specific financial support.

### Time-to-completion of transfer degree

RiSE students (Fig 1B) spent an average of 10 quarters (more than three academic years) at Edmonds College, despite being a “two-year” college. They also stayed "at risk" to complete a degree longer than the Not-RiSE^C^ students (Fig 7). This may be because they persisted and did not drop out as early as the Not-RiSE^C^ students, or they were more likely than the comparable population to return to complete a degree.

### Student intent

Identifying STEM transfer students and what field of study they originally intended to pursue when they first enrolled in college is complicated. Over 30% of the RiSE students changed their “intent” (i.e. academic track) at least once over the course of their enrollment at EC. Of those who switched intent, 51% of students’ second intents changed to academic transfer. Most of the students changing to transfer intent were students who had first enrolled at the college for developmental education or job skills purposes. This reflects a net gain of students joining the STEM transfer pipeline, instead of a net loss or “leak” in the “STEM pipeline.” In contrast to the common “pipeline” metaphor, more recent research focuses on “momentum” and “momentum trajectories.” Wang [53] describes four “momentum trajectories” for community college students intending to transfer: linear upward (40%), detoured (13%), deferred (9%), taking a break (38%). Students on the latter three of these four trajectories may spend several years in community colleges as reflected in our data in Fig 1B (with some students enrolled for more than 20 quarters) and Fig 7, which shows smaller, later peaks in time-to-degree. Students’ extended time in the community college environment may result in them joining the STEM transfer pathway during their community college education. This delayed entry into STEM transfer intent has not been well-characterized and may provide an untapped opportunity for increasing the STEM transfer population.

RiSE students did not have higher persistence because they had more resources; in fact, they were more financially disadvantaged compared to their not-RiSE^C^ peers (Fig 2). The RiSE program was designed to cultivate persistence through belonging to a community and providing access to resources, regardless of financial disadvantage. These results are consistent with other evidence that institutional changes to the students’ environment that include implementation of learning communities, orientations, and academic support can help increase persistence [54]. It also has been shown that creating a supportive community for STEM students helps all students persist and can help close the achievement gap for underrepresented students [29].

Lack of academic momentum is often seen as a barrier to undergraduate student success, and reports have suggested that this is more of a challenge at community colleges [26–28,55]. Nontraditional-aged community college students might have several academic interruptions that may or may not have to do with academic motivation, ability, or preparation; their momentum may be hindered because they work full-time to support themselves or others, are care providers (for children, siblings, parents or others), and are less likely to have financial support, health care or stable housing [27,28]. These challenges may result in setbacks regarding academic success (perhaps a grade of D, F or W in a gateway STEM course) and persistence (missing one or more academic terms) over the time period usually considered typical for completion of an undergraduate degree (4–6 years). Our data demonstrate that some students can progress and succeed in STEM courses despite a loss of academic momentum, perhaps progressing at a slower pace and with interruptions. RiSE students who failed a gateway math course still had a higher probability of persistence in the long run than comparable Not-RiSE^C^ students who passed the same courses with a B average (Fig 4A and 4B). Also, a higher percentage of RiSE students (34%) completed an associate degree at Edmonds College compared to Not-RiSE^C^ students (27%). Furthermore, some RiSE students completed associate degrees 8–10 years after their initial enrollment at the college (Fig 7). RiSE provided support from faculty and peers and offered resources for students through early failure and disruption in academic momentum. We suggest that supporting students through early failure and academic interruptions can result in long-term academic persistence and success, which has the potential to increase the diversity and resilience of the STEM community and workforce.

### Limitations

These data reflect a post-hoc analysis of data from a STEM student program at a single, suburban two-year college in the Pacific Northwest. These students may be different in many ways from other populations of undergraduate STEM students, so the results must be interpreted with caution. This was not an experimental study and there was no single randomized control group with which to compare all of the data. Furthermore, the RiSE program did not involve a single, discrete intervention, rather it was a program designed to increase relationships between students, faculty, and staff to create a supportive community. Therefore, conclusions cannot be drawn about specific elements of the RiSE program.

Many of the support and enrichment elements were not exclusive to only RiSE students, although participation was much higher for RiSE students due to the targeted promotion of the events through RiSE emails and social media. RiSE students were required to participate in a RiSE event and academic advising each academic quarter. RiSE students also were contacted regularly by STEM staff or faculty and encouraged to participate, but the ways and amount to which individual students experienced and participated in RiSE varied significantly.

A few limitations should be noted. First, Edmonds College had higher pass rates (lower DFW rates) in STEM courses than those reported in the literature from 4-year universities. Freeman [22] cited data from the early 2000s showing that it was common for one-third of students to fail in STEM gateway courses, including calculus and general chemistry. This is consistent with a 2018 Gardner Institute study of 36 colleges and universities (including seven community colleges) that reported an average DFW rate of 29% for introductory Chemistry and 34% for introductory calculus [56]. Edmonds College DFW rates (Table F1 in S1 File) for Calculus (24%) are lower than those reported in the literature and the EC DFW rates for General Chemistry are much lower (16–19%). The higher passing rates (lower DFW rates) at EC may make our results less robust to generalization. It is important to note that DFW information in the literature typically reflects courses at research intensive institutions, and we do not know if our data are more representative of community college data in general. However, the inclusion of community colleges in the Gardner Institute study does not support the suggestion that community colleges have lower DFW rates in STEM gateway courses.

Second, lack of identification of STEM majors is a common limitation at two-year colleges. Edmonds and other two-year colleges generally do not collect information on students’ intended majors and advising is usually not mandatory. This results in a lack of identification of students who would identify as STEM students among the general student population. Therefore, although overall student persistence can be tracked, retention within a major or a STEM pathway cannot.

Finally, another complicating factor is that Edmonds College is just one of 18 community/technical colleges within the Seattle-Tacoma metropolitan area and one of 34 two-year colleges in the state. Many students move among these two-year colleges to complete their prerequisites for transfer and/or associate degrees. Therefore, actual persistence of students in college and in STEM gateway sequences may be higher than reported here. Also, students in STEM programs may complete their prerequisites at a two-year college and transfer to a four-year program without an associate degree [57,58].

### Implications for STEM programs and departments

This study contributes to the research literature focused on community college STEM students, a population underrepresented in the STEM education research literature. Our data should be useful to all faculty and support staff at all colleges and universities interested in retaining STEM students through initial years of higher education STEM courses.

Because the college is small compared to research universities, implementation of the RiSE program required involvement by the majority of full-time and many of the part-time faculty in the STEM division. Faculty and staff were essential to implementing the student support elements, including a core team (eight faculty from different STEM departments), a STEM study room (21 faculty), and specific student events including quarterly advising (19 faculty), STEM quarterly kickoffs (25 faculty), and STEM awards (37 faculty) from of a STEM division that includes only 37 full-time faculty and approximately the same number of part-time faculty.

We cultivated a STEM community and provided resources to support students, and we recommend that other institutions do this intentionally as well. Multiple opportunities should be given for students to engage with peers, faculty, and resources, and to form relationships and build a community. For the RiSE program that included the creation of a STEM study room that provided peer tutors, faculty, computers, refreshments, and multiple opportunities for academic and social interactions. Although initially supported by grant funding, the STEM staff person has continued to be funded as a division staff position as an “embedded” advisor in the STEM division. The success of the embedded advisor model led to similar positions in other divisions at the college. The peer tutors are an ongoing expense in the STEM study room. Post-grant funding for these tutors has come from a few sources: student government, the college’s foundation, and supplemental instruction through the STEM division. The STEM study room is also supported through STEM faculty holding “office hours” in that space. Although the cultural shift by faculty to hold office hours in the STEM study room was not immediate, it has become more the norm, allowing the room to be open and staffed every weekday for the bulk of the day.

As we have seen, students who are financially disadvantaged or who temporarily lose momentum can be successful when supported. An encouraging community can help increase their confidence and allow them to believe they can succeed. Some may not start on the pathway to earn a transfer degree in STEM, but with encouragement and increased confidence they may change their path or “intent” to STEM transfer. Educators and administrators should understand that students may need additional time to complete a degree. Students may experience early failure or a loss of momentum due to personal circumstances, but many will retake classes and persist through to degree attainment if given the time and support. Students also may need assistance from the community to navigate financial aid or other institutional barriers if they struggle academically. Experience and comfort with failure is necessary in many STEM fields, and our students have experience learning from and persisting through failure to achieve success.

Based on our experience with and data from the RiSE program, we recommend intentionally cultivating a community of STEM students and providing students with support and time to persist and be successful. We recommend leveraging existing resources, particularly at under-resourced two-year colleges, to create this support. For example, explore existing tutoring programs (e.g., peer instruction) and personnel resources (advising, office hours, services learning, and undergraduate research opportunities). Furthermore, we encourage STEM faculty and departments to do this in their current state. Do not wait for more external resources, administrative continuity, and top-down leadership. You can begin now to provide the following support for students:

- academic support in the form of peer tutors, classmates, faculty, and staff.
- structured academic advising and formal and informal mentoring (in office hours, at events, through service learning, and with undergraduate research).
- multiple formal and informal opportunities for students to engage with other students, faculty, and resources.
- a student-centered space (e.g., the STEM study room).
- time, to navigate potential “time to degree” barriers and financial aid challenges, to allow the ability to retake STEM gateway courses and to change their goals and intent to a STEM discipline.

## Supporting information

S1 File Additional information, definitions, and data tables. (DOCX)
