# Supplementary material for: Cultivating a Science, Technology, Engineering and Mathematics (STEM) community for two-year college student success and persistence
Source: PLoS One. 2023 Sep 8;18(9):e0290958. doi: 10.1371/journal.pone.0290958 (PMC10490953; doi:10.1371/journal.pone.0290958)
Supplement: S1 File — (DOCX) [file pone.0290958.s001.docx]

S1_File: Supporting Information

# Table of Contents page

**Appendix A.** Measures of Persistence, Definitions and Data

**Table A1.** Definitions of measures of persistence 1

**Appendix B.** Comparison Populations

**Table B1.** Populations used to compare to the RiSE student population 2

**Appendix C.** Formation of a Not-RiSE^C^ Comparable Population 3

**Table C1.** Academic indicators in identification of Not-RiSE^C^ comparable population

**Table C2.** Summary estimated regression coefficients for propensity model

**Figure C1.** Propensity model averaged importance of indicators

**Appendix D.** Persistence in gateway Math and Chemistry courses sequences 7

**Table D1.** Persistence in Gateway Math Courses

**Table D2.** Persistence in Gateway General Chemistry Courses

**D3.** Explanation of course persistence calculations

**Appendix E.** GPA in top-10 STEM courses 13

**Table E1.** Example of confounding variables for two terms of Precalculus 2

**Appendix F.** DFW Rates in Gateway STEM courses 14

**Table F1.** Average Rates of DFW (non-passing marks of D or F grades and Withdrawals) for mathematics and chemistry gateway courses

# Appendix A. Measures of persistence & Definitions

A few different measures of student persistence were used in this study. Table A1 is a guide to these measures of persistence and the data figures and tables in which these were used.

**Table A1.** Definitions of measures of persistence

| Persistence measures | Definition | Relevant Figures & Tables |
| --- | --- | --- |
| quarter-to-quarter persistence rate | The fraction of students in a specific academic term that were enrolled in the following academic term (excluding those who completed degrees or certificates) | Figure 3, median persistence rates |
| persistence probability | A student’s probability of enrolling in the next course in a sequence, relative to that student’s grade in their first attempt at the preceding course in the sequence. | Figures 4A & 5A, persistence probability |
| persistence probability ratio | The ratio of RiSE vs. Not-RiSE^C^ student persistence probability, given the student’s grade in their first attempt at the preceding course in the sequence. | Figures 4B & 5B, persistence probability ratios |
| course persistence ratio | The course persistence ratio is the ratio of the populations (RiSE/Not-RiSE^C^) represented in each course persistence pair. | Appendix E, Tables E1 & E2 in S1_File |

# Appendix B. Comparison Populations

Data from the students in the RiSE population (RiSE) were compared with other groups of students who were not in the RiSE Program (Not-RiSE). Multiple comparison groups were used because a single control group was not possible or appropriate. Also, different comparison groups were helpful to answer different questions being asked at the institution, division, and program levels. The different Not-RiSE comparison populations and the figures in which they are used are summarized in Table B1.

**Table B1.** Populations used to compare to the RiSE student population

| Comparison population | Description | Relevant Figures |
| --- | --- | --- |
| Not-RiSE^A^ | The mean GPAs of equal-size random samples of RiSE students and students not in RiSE were drawn from a randomly chosen top-10 STEM course in a randomly-chosen quarter. This was done 100,000 times. | Figure 6, GPAs in top-10 STEM Courses |
| Not-RiSE^B^ | This comparison population included students at the college who were in the same classes as RiSE students (all classes, not just STEM). | Figure 3, quarter-to-quarter persistence rates |
| Not-RiSE^C^ | This is the comparable (or matched) population of students, formed by a propensity score process, who were very similar to RiSE students as described in detail in the Supporting Information, Appendix A in S1_File. | Figure 2, financial disadvantage  Figures 4 & 5, course persistence  Figure 7, degree completion |

# Appendix C. Formation of a Not-RiSE^C^ Comparable population

To form a comparable population, a random sample was drawn of N=10,000 students who were not in the RiSE program who were enrolled over the period of first known RiSE student enrollment (Fall 1995) through the Spring 2017. Students’ first-math and first-English courses, and their associated grades, were used as proxy measures of student preparedness for college work. Therefore, students who had not taken at least one math course and one English course at Edmonds College (formerly Edmonds Community College, EdCC) were dropped from the sample of 10,000. The remaining candidate comparable population size was then N=6634 students who had taken both at least one math course and at least one English course at Edmonds College.

The next step at identification of a Not-RiSE^C^ comparable population was to assume a set of 18 academic indicator variables that existed in student records at the college that we believed would have relevance to the academic outcomes of time-to-completion of degree, rate of degree attainment, and STEM population diversity. These indicators were either taken or calculated from campus transcript and student demographic databases.

In addition to the four first-math and first-English (course and grade) indicators, 14 additional indicator variables in the campus database of student transcript and demographic data were chosen, including: student age (on Pi-Day, 03/14/2016), binary gender identification, first expressed intent for enrolling at the college (e.g., for an academic transfer degree, certificate, developmental ed., etc.), first-reported IPEDS degree-seeking indicator, first reported disability status, first reported status of economic disadvantage, family status, full-time/part-time student status, kind-of-student (transfer/workforce/basic skills/other), prior education, race, running start status, and work status. Using these 18 indicators, an initial candidate binary logistic regression model was formed then passed to the R package gmulti to select the most parsimonious submodel via genetic search algorithm to locate submodels based on Akaike information criterion (AIC). The search- stopping/convergence criteria were arbitrarily chosen to be when no further changes in best individual model AIC were greater than 0.05 and no changes in mean AIC were greater than 0.7 for at least 5 consecutive generations. Convergence was attained after 480 generations. The most parsimonious sub-model based on reduction in Akaike information resulted in dropping 7 potential indicator variables from the model (degree-seeking, disability, family status, full/part time status, health limitation, running start status, work status). Figure C1 summarizes the model-averaged relative importance of each of the 18 originally-chosen RiSE student propensity indicators. The informative indicators were retained in the final propensity model (importance 0.80), as the variables (e.g., disability status, full/part time status, family status, etc.) were shown to be relatively uninformative regarding RiSE student identification. The final propensity score model of RiSE membership retained the other 11 indicators shown in Table C1 and Figure C1. The R MatchIt package was then used with the reduced 11 indicators glm propensity score model to obtain matched RiSE and Not-RiSE^C^ populations via genetic algorithm with genetic population size of N=10,000. The final matched population sizes consisted of N(Not-RiSE^C^)=260 Not-RiSE^C^ students and N(RiSE)=294 RiSE students with no standardized mean population indicator differences exceeding 0.10.

**Table C1.** Academic indicator variables considered in identification of Not-RiSE^C^ comparable population

| **Indicator** (variable code) | **indicator variable description** | **Retained*** | **Dropped** |
| --- | --- | --- | --- |
| AGE.PI | Student age on Pi-Day, 03/14/2016 | X |  |
| SEX.2 | Gender identification for the first quarter of enrollment at Edmonds College: female or male (binary) | X |  |
| ECON_DISAD_IND.1 | Qualified for financial aid for the first quarter of enrollment at Edmonds College (yes or no) | X |  |
| INTENT.1 | First expressed intent for the first quarter of enrollment at Edmonds College (e.g., for an academic transfer degree, certificate, developmental ed., etc.) | X |  |
| KIND_OF_STUDENT.1 | Kind-of-student (transfer/workforce/basic skills/other), during first quarter of enrollment. | X |  |
| PRIOR_EDUC.1 | Years of education before enrollment at Edmonds College, for the first quarter of enrollment at the college (e.g., 13 for someone who had completed a year of post-secondary education elsewhere) | X |  |
| RACE_CODE.1 | American Indian or Alaska Native (AIAN), Asian, Black or African American (BAA), Hispanic or Latino, Mixed, Native Hawaiian or Pacific Islander (NHPI), White, Other | X |  |
| M1.COURSE | First MATH course taken at at Edmonds College | X |  |
| M1.GR_DEC | Grade in first MATH course taken at Edmonds College | X |  |
| E1.COURSE | First English (ENGL) course taken at Edmonds College | X |  |
| E1.GR_DEC | Grade in first English (ENGL) course taken at Edmonds College | X |  |
| DEGREE_SEEK.1 | First-reported IPEDS degree-seeking indicator (yes or no) |  | X |
| FULL_PART_TIME_IND.1 | Full- time or part-time student status for the first quarter of enrollment at Edmonds College (binary) |  | X |
| RUNNING_START_STATUS.1 | Running Start (also enrolled in high school) during the first quarter of enrollment at Edmonds College (yes or no) |  | X |
| WORK_ATTEND.1 | Work Status for the first quarter of enrollment at Edmonds College (28 possible numeric codes) |  | X |
| FAM_STAT.1 | Family Status for the first quarter of enrollment at Edmonds College (7 possible alphanumeric codes) |  | X |
| HEALTH_LIM.1 | Health Status for the first quarter of enrollment at Edmonds College (19 possible alphanumeric codes) |  | X |
| DISABILITY.1 | First reported disability status (yes or no) |  | X |

*Of the eighteen student demographic and academic variables used as propensity model indicators, eleven were retained by the model-selection process.



**Figure C1.** Model-averaged relative importance (MARI) of all 18 available indicators initially chosen for the general linear model (GLM) classification of RiSE vs. Not-RiSE^C^ students. The 11 most-informative indicators distinguishing RiSE from Not-RiSE^C^ students are taken as those indicators with MARI>=0.80, which are then used in propensity scoring step to identify the subset of Not-RiSE^C^ students which most resemble the RiSE students balanced on these 11 indicators. See Burnham and Anderson (2002) for details motivating this choice of model selection. Some of the potential indicators, including disability status, full-time or part-time status, family status, were relatively uninformative about RiSE student identification.

Burnham KP Anderson DR. *Model Selection and Multimodal Inference: A Practical Information-Theoretic Approach*. 2nd ed. New York: Springer; 2002:149-205.

**Table C2.** Summary of estimated regression coefficients for propensity model

| **Indicator** (“BASE.VAL”)VAL | **EST** | **SE** | **Z** | **P** |
| --- | --- | --- | --- | --- |
| E1.COURSE (“ENLG&101”)ENGL099 | -3.47 | 1.04 | -3.34 | 0.00 |
| E1.COURSE (“ENLG&101”)ENGL205 | -2.54 | 1.04 | -2.45 | 0.01 |
| E1.COURSE (“ENLG&101”)ENGL105 | -2.02 | 0.33 | -6.08 | 0.00 |
| E1.COURSE (“ENLG&101”)ENGL&102 | -0.96 | 0.41 | -2.33 | 0.02 |
| E1.COURSE (“ENLG&101”)ENGL100 | -0.78 | 0.17 | -4.74 | 0.00 |
| M1.COURSE (“MATH&141”)MATH140 | -2.25 | 0.73 | -3.06 | 0.00 |
| M1.COURSE (“MATH&141”)MATH040J | -1.77 | 0.75 | -2.34 | 0.02 |
| M1.COURSE (“MATH&141”)MATH087 | -1.73 | 0.62 | -2.80 | 0.01 |
| M1.COURSE (“MATH&141”)MATH077 | -1.52 | 0.64 | -2.39 | 0.02 |
| M1.COURSE (“MATH&141”)MATH&146 | -1.03 | 0.43 | -2.38 | 0.02 |
| M1.COURSE (“MATH&141”)MATH097 | -0.98 | 0.47 | -2.10 | 0.04 |
| M1.COURSE (“MATH&141”)MATH080 | -0.51 | 0.24 | -2.10 | 0.04 |
| M1.COURSE (“MATH&141”)MATH&151 | 0.52 | 0.23 | 2.26 | 0.02 |
| M1.COURSE (“MATH&141”)MATH&152 | 1.59 | 0.57 | 2.82 | 0.00 |
| M1.COURSE (“MATH&141”)MATH155 | 2.52 | 0.60 | 4.17 | 0.00 |
| INTENT.1 (“B” Transfer) D – Adult Basic Education | 1.03 | 0.53 | 1.95 | 0.05 |
| INTENT.1 (“B” Transfer) E – Developmental Education | 2.78 | 0.37 | 7.56 | 0.00 |
| KIND_OF_STUDENT.1 (“T” Transfer)B – Basic Skills | -1.41 | 0.52 | -2.69 | 0.01 |
| AGE.PI | -0.06 | 0.01 | -4.82 | 0.00 |
| SEX.2 (“F” Female)M – Male | 0.28 | 0.13 | 2.13 | 0.03 |
| E1.GR_DEC | 0.16 | 0.07 | 2.38 | 0.02 |
| M1.GR_DEC | 0.25 | 0.06 | 4.01 | 0.00 |
| ECON_DISAD_IND.1 (“N”)Y | 0.89 | 0.17 | 5.18 | 0.00 |
| PRIOR_EDUC.1 (“13” HS grad)14 – Some post-HS | 0.76 | 0.20 | 3.77 | 0.00 |
| PRIOR_EDUC.1 (“13” HS grad)12 – GED | 1.04 | 0.31 | 3.32 | 0.00 |
| PRIOR_EDUC.1 (“13” HS grad)90 – Other | 1.36 | 0.38 | 3.59 | 0.00 |
| RACE_CODE.1(“6” White)2 – African American | 0.68 | 0.27 | 2.50 | 0.01 |
| RACE_CODE.1(“6” White)0 – Other Race | 0.92 | 0.40 | 2.32 | 0.02 |
| RACE_CODE.1(“6” White)999 – Blank Not Reported | 0.93 | 0.22 | 4.21 | 0.00 |
| RACE_CODE.1(“6” White)3 – American Indian | 1.83 | 0.53 | 3.45 | 0.00 |

Table C2 shows the summary of the estimated regression coefficients (EST), their standard errors (SE), coefficient Wald test statistics (Z), and the p-values indicating significant differences from zero for the regression coefficients. For example, in row 1 below, the log-odds of being a RiSE student is reduced by -3.47 if a student’s first English course (E1.COURSE) was ENGL099, relative to a ”base-line” student whose first English course was ENGL&101. Similarly, each unit GPA increase in a student’s first math course (M1.GR DEC) increases their log-odds of being a RiSE student by 0.25.

# Appendix D. Persistence in gateway Math and Chemistry courses sequences

Observations:

Observations in Table D1 and Table D2 are the numbers of students the RiSE and Not-RiSE^C^ populations for each course pair.

Log-Likelihood and Effects Size:

The Log-Likelihood in persistence Table D1 and Table D2 refer to the “feasibility” or “explanatory capability” of the model which takes into account grades Gm and RiSE membership (R) simultaneously, relative to an “empty model” where only gross persistence rates (characterized by the coefficient β_0_) is taken into account.

$G_{m}$ is the decimal grade $G_{m}\in\left[0.0,4.0\right]$that a student received in course $C_{m}$. In regression Tables D1 and D2, β_m_ refers to the value of the coefficient of the variable $G_{m}$. The coefficient β_m_ as it appears in the overall fitted model

$z\text{ }= \text{ }\beta_{0}+ \beta_{m}G_{m}+ \beta_{R}R\text{ }$ (1)

is an effect size indicating the effect of receiving grade $G_{m}$ in course $C_{m}$ on the log-odds of arrival in course $C_{n}$ from the course $C_{m}$, in the presence of information about RiSE membership.

We have a data set D that includes measurables $\text{M=}\left(G_{m},R\right)$. We assumed that both grade $G_{m}$ and RiSE membership, $R$, are potential predictors of success for progressing from course $C_{m}$ to course $C_{n}$ , and that we can model the log-odds of this progression as

$z\text{ }= \text{ }\beta_{0}+ \beta_{m}G_{m}+ \beta_{R}R\text{ }$ (2)

We proceeded by estimating the most-probable values of the coefficients $\beta=\left(\beta_{0}, \beta_{m},\beta_{R}\right)$that appeared to have the greatest likelihood of having the data $D$ being generated by the assumed model $z\left(\beta_{0}, \beta_{m},\beta_{R}\right)$. For this purpose, we computed the Log-Likelihood of two competing candidate models: $z\left(\beta_{0}, \beta_{m},\beta_{R}\right)$ (grades and RiSE simultaneously matter) versus $z\left(\beta_{0}\right)$ (grades and RiSE are simultaneously irrelevant). To do so, we calculated the distribution of values of alternative parameter sets $\beta=\left(\beta_{0}, \beta_{m},\beta_{R}\right)$and choose the one that maximized the log-likelihood of the data set $D$ having arisen from it. This is the Log-Likelihood of our *specific* model:

$LL\left(D\left|\beta\right.\right)$ (3)

Next, we calculated the log-likelihood that the parameters $\beta=\left(\beta_{0}, 0,0\right)$ explained the data $D$. This is the log-likelihood of the data $D$ arriving from an “empty” model, $LL\left(D\left|\beta=\left(\beta_{0}, 0,0\right)\right.\right)$.

The quantity

$LLR=-2\left[LL\left(D\left|\left(\beta_{0}, 0\right)\right.\right)-LL\left(D\left|\beta\right.\right)\right]$ (4)

which represents (−2) times the log of the likelihood ratio (LLR) of the assumed model vs. the empty model is a chi-squared distributed statistic with d = 2 degrees of freedom. We used a table of the chi-squared distribution and determined the significance of realizing our data $D$ from our model $z\left(\beta\right)$ as a random effect relative to (grades + RiSE) not mattering at all. In the case of MATH090 →MATH141 persistence, we have that

$LLR=-2\left[LL\left(-1.679, 0,0\right)-LL\left(-1.679, 0.577,2.739\right)\right]=-97.672$ (5)

From the the chi-squared distribution table, the probability of observing a result larger than 97.672 for a chi-squared distributed statistic is

$p\left(LR\geq97.762\right)=6.2\times10^{-22}<<0.001$ (6)

Course Persistence Ratio:

The Course Persistence Ratios are the ratios of the populations (RiSE/Not-RiSE^C^) represented in each course persistence pair. For example, the number of RiSE students in the comparable population sample who progressed from Math&141 to Math&142 is 1.78 times the number of Not-RiSE^C^ students in the comparable population sample who progressed from Math&141 to Math&142.

**Table D1.** Persistence in Gateway Math Courses: Likelihood of arrival in the next course in the MATH sequence (*C_n_*), based on grade (*G_m_*) in prior course (*C_m_*) and RiSE participation. This table contains maximum likelihood estimates of the logistic regression model parameters given the data, for the model used for Figures 4A & B.

|  | MATH 090 to MATH&141 | MATH&141 to MATH&142 | MATH&142 to MATH&151 |
| --- | --- | --- | --- |
| Observations:  total (RiSE)  [RiSE % of total] | 233 (122)  [52% RiSE] | 333 (213)  [64% RiSE] | 262 (192)  [73% RiSE] |
| β*_m_*, Effect size of grade (G_m_) in C_m_ | 0.577***  (0.286, 0.867)^3^ | 0.584***  (0.382, 0.787)^3^ | 0.665***  (0.414, 0.916)^3^ |
| ^1^β_R_, Effect size of RiSE participation | 2.789***  (1.900, 3.579)^3^ | 1.997***  (1.393, 2.601)^3^ | 1.188***   (0.468, 1.909)^3^ |
| ^2^β_0_, Effect size of presence in C*_m_* alone | 1.697***   (-2.613, 0.78)^3^ | -1.472***  (-2.173, 0.770)^3^ | -0.868**  (-1.703, -0.032)^3^ |
| Log-Likelihood | -97.762 | -147.730 | -106.092 |
| Course Persistence Ratio | 1.10 | 1.78 | 1.13 |

**Notes:**

^1^We interpret the coefficient β_R_ as the comparative advantage in course persistence of RISE relative to Not-RiSE^C^ students.

^2^We interpret the coefficient β_0_ as the log-odds of a student's arrival in C_n_, without regard to RiSE participation or their grade (G_m_) in course C_m_.

^3^95% confidence intervals for coefficients β*_m_*, β_R_, & β_0_ are shown in parenthesis.

*p<0.1; **p<0.05; ***p<0.01

**Table D2.** Persistence in Gateway General Chemistry Courses: Likelihood of arrival in the next course in the CHEM sequence (*C_n_*), based on grade (*G_m_*) in prior course (*C_m_*) and RiSE participation. This table contains maximum likelihood estimates of the logistic regression model parameters given the data, for the model used for Figures 5A & B.

|  | CHEM&139 to CHEM&161 | CHEM&161 to CHEM&162 | CHEM&162 to CHEM&163 |
| --- | --- | --- | --- |
| Observations:  total (RiSE)  [RiSE % of total] | 204 (155)  [76% RiSE] | 242 (189)  [78% RiSE] | 162 (129)  [80% RiSE] |
| β*_m_*, Effect size of grade (G_m_) in C_m_ | 0.629***  (0.374, 0.883)^3^ | 0.393***  (0.374, 0.883)^3^ | 0.364**  (0.083, 0.645)^3^ |
| ^1^β_R_, Effect size of RiSE participation | 1.320***  (0.544, 2.096)^3^ | 0.265  (-0.390, 0.920)^3^ | -0.880**   (-1.737, -0.023)^3^ |
| ^2^β_0_, Effect size of presence in C*_m_* alone | -1.193***   (-2.081, -0.305)^3^ | -0.569  (-1.396, 0.258)^3^ | -0.068  (-1.181, 1.045)^3^ |
| Log-Likelihood | -90.661 | -146.924 | -105.309 |
| Course Persistence Ratio | 3.16 | 3.57 | 3.91 |

**Notes:**

^1^We interpret the coefficient β_R_ as the comparative advantage in course persistence of RISE relative to Not-RiSE^C^ students.

^2^We interpret the coefficient β_0_ as the log-odds of a student's arrival in C_n_, without regard to RiSE participation or their grade (G_m_) in course C_m_.

^3^95% confidence intervals for coefficients β*_m_*, β_R_, & β_0_ are shown in parenthesis.

*p<0.1; **p<0.05; ***p<0.01

**D3.** Explanation of course persistence calculations for Gateway STEM Course Progressions

To examine course (C_m_) to course persistence (C_n_) in a STEM course sequence (e.g., precalculus) we used a logistic regression method to predict the log-odds of arrival in the second course (C_n_).

In this model,$C_{m}$ was a course that a student was registered in at time $t_{m}$, and $C_{n}$ was a later course, $C_{m}\prec C_{n}$ that they were registered in at time $t_{m}<t_{n}$. We assume a time-ordered dependency that the probability of arrival in $C_{n}$ from $C_{m}$ is (1) proportional to the grade, $G_{m}$, that the student received in course $C_{m}$and is (2) stratified according to RiSE group membership, $R\in\left\{0,1\right\}$, with a student either being a member of RiSE (R = 1) or not a member of RiSE (R = 0). We assume a simple functional relationship between the log-odds of "arrival" in course $C_{n}$ of the form

(1)

$=\beta_{0}+\beta_{m}G_{m}+\beta_{R}R$

Where $p\left(\left.A_{n}\right|G_{m},R\right)$ is the probability of arrival in course $C_{n}$, given grade $G_{m}$received in course$C_{m}\prec C_{n}$ and RISE membership, $R$. We interpret the parameter $\beta_{0}$ as the log-odds of a Not-RiSE^C^ (R=0) student's arrival in $C_{n}$ given that they received a grade of $G_{m}=0$ in their first-attempt at course $C_{m}$,

$z\left(\left.A_{n}\right|0,0\right)=\ln\left(\frac{p\left(\left.A_{n}\right|0,0\right)}{1-p\left(\left.A_{n}\right|0,0\right)}\right)=\beta_{0}$ (2)

The probability of such a ($G_{m}=0$;$R=0$)-type (Not-RiSE^C^) student later "arriving" in course $C_{n}$ is then

$p\left(A_{n}\left|0,0\right.\right)=\frac{e^{\beta_{0}}}{1+e^{\beta_{0}}}$ (3)

A ($G_{m}=0$;$R=1$)-type student is a RiSE student. The log-odds of arrival in the same course $C_{n}$ for a RISE student is

$z\left(A_{n}\left|0,1\right.\right)=\beta_{0}+\beta_{R}$ (4)

The coefficient

$\beta_{R}=z\left(A_{n}\left|G_{m},1\right.\right)-z\left(A_{n}\left|G_{m},0\right.\right)$ (5)

$=\ln\left[\frac{\left(\frac{p\left(A_{n}\left|G_{m},1\right.\right)}{1-p\left(A_{n}\left|G_{m},1\right.\right)}\right)}{\frac{p\left(A_{n}\left|G_{m},0\right.\right)}{1-p\left(A_{n}\left|G_{m},0\right.\right)}}\right]$ (6)

represents the log-odds-ratio of a RiSE student with grade $G_{m}$in course $C_{m}$arriving in course $C_{n}$relative to a Not-RiSE^C^ student with grade $G_{m}$arriving in course $C_{n}$. This particular measure characterizes the difference between RISE and Not-RiSE^C^ student course transitions $C_{m}\to C_{n}$among students who received the same grade $G_{m}$ for their first attempt at course $C_{m}$ before eventually arriving (or not) in course $C_{n}$.

The coefficient $\beta_{R}$ is a measure of comparative advantage in course persistence of RISE students relative to Not-RiSE^C^ students. If $\beta_{R}=0$, then there is no comparative persistence advantage; if $\beta_{R}>0$, then RISE students have a comparative persistence advantage over Not-RiSE^C^ students; if $\beta_{R}<0$, then Not-RiSE^C^ students have a comparative persistence advantage over RISE students.

We can translate the comparative advantage effects size $\beta_{R}$ into an effective course grade advantage by comparing the differential increase (or decrease) in grade points $\delta G_{m}$ required by Not-RiSE^C^ students in course $C_{m}$to have the same persistence probability of RISE students. The log-odds ratio of RISE student persistence given grade $G_{m}$ at any grade point level relative to Not-RiSE^C^ students given grade ($G_{m}+\delta G_{m}$) is

$=\ln\left(\frac{z\left(A_{n}\left|G_{m},1\right.\right)}{z\left(A_{n}\left| \left(G_{m}+\delta G_{m}\right),0\right.\right)}\right)=\beta_{R}-\beta_{m}\delta G_{m}$ (7)

Thus, for any fixed grade $G_{m}$, a differential increase (decrease) of

$\delta G_{m}=\frac{\beta_{R}}{\beta_{m}}$ (8)

grade points by Not-RiSE^C^ students is required to make the comparative persistence advantage of Not-RiSE^C^ students equal to that of RISE students. For example, using the fitted coefficients for the course transition $C_{m}$= MATH090$\to C_{n}$ = MATH&141 transitions for the matched populations,

$\begin{matrix} \beta_{0}=-1.697 \\ \beta_{m}=0.577 \\ \beta_{R}=2.740 \end{matrix}$ (9)

the differential grade increase in MATH090 required by a Not-RiSE^C^ student to have the same probability of persisting to MATH&141 as a RISE student at the same grade level is $\delta G_{m}=4.8$. In other words, even a Not-RiSE^C^ student with a grade of 4.0 for their first-attempt in MATH090 has a lesser probability of persisting to MATH&141 than does a RISE student coming from MATH090, including those RISE students who received a grade of zero on their first MATH090 attempt.

Likewise, comparing $C_{m}$= MATH&141$\to C_{n}$= MATH&151 transitions, we have

$\begin{matrix} \beta_{0}=-0.868 \\ \beta_{m}=0.665 \\ \beta_{R}=1.189 \end{matrix}$ (10)

which indicate that a Not-RiSE^C^ student would need an additional $\delta G_{m}=1.8$ grade points to be as likely to persist to MATH&151 as is a RISE student at any MATH&141 grade level$G_{m}$, i.e., a RISE student with a first-attempt MATH&141 grade of $G_{m}$= 1.0 is as likely to persist to MATH&151 as is a Not-RiSE^C^ student with a first-attempt MATH&141 grade of $G_{m}$= 3.8.

From the fitted coefficients, we can determine the probability of arrival $A_{n}$ from course $C_{m}$ as a function of course grade $G_{m}$and RISE membership, *R*,

$p\left(A_{n}\left|G_{m},R\right.\right)=\frac{e^{\beta_{0}+\beta_{m}G_{m}+\beta_{R}R}}{1+e^{\beta_{0}+\beta_{m}G_{m}+\beta_{R}R}}$ (11)

as in Figure 4A, as well as the persistence ratio

$\frac{p\left(A_{n}\left|G_{m},1\right.\right)}{p\left(A_{n}\left|G_{m},0\right.\right)}=\frac{e^{\beta_{R}}\left(1+e^{\beta_{0}+\beta_{m}G_{m}}\right)}{1+e^{\beta_{R}}e^{\beta_{0}+\beta_{m}G_{m}}}$ (12)

as in Figure 4B.

# Appendix E. GPA in top-10 STEM courses

It is difficult to ask the question "How does the mean GPA of RISE students compare to that of the students who were not in RiSE in the top-10 STEM courses?”, because the answer depends simultaneously on several confounding variables:

(1) the course,

(2) the quarter,

(3) the number of RISE of students in the course, and 
(4) the number of Not-RISE students in the course.

The answer to this question will change depending on which course and which quarter of each course one chooses.

For example, Table D1 shows that for Pre-Calculus 2 (MATH& 142) in Fall quarter 2016 (B672): N(RiSE)=8, N(Not-RiSE^A^)=148, RiSE mean =2.55, Not-RiSE^A^ mean GPA =2.59) vs. the same course (MATH &142) in Spring 2012 (B124): N(RiSE)=11, N(Not-RiSE^A^)=253, RiSE mean GPA= 2.86, Not-RiSE^A^ mean GPA =2.11). Due to the typical non-normality of the grade distributions (these fail Shapiro-Wilk tests), one cannot legitimately do a two-sample t-test to determine whether or not the GPAs differ.

**Table E1.** Example of confounding variables for two terms of Precalculus 2 (MATH& 142)

|  | **RiSE**  **(N)** | **Not-RiSE**^A^  **(N)** | **RiSE**  **mean GPA** | **Not-RiSE**^A^  **mean GPA** |
| --- | --- | --- | --- | --- |
| Spring 2012 (B124) | 11 | 253 | 2.55 | 2.59 |
| Fall quarter 2016 (B672) | 8 | 148 | 2.86 | 2.11 |

Instead, of addressing the question "How does the mean GPA of RISE students compare to that of the Not-RISE^A^ students in the top-10 STEM courses?”, we reframed it to ask: "If we take equal-size random samples of RiSE and Not-RiSE^A^ students, drawn from a randomly-chosen top-10 STEM course in a randomly-chosen quarter and calculate the mean GPAs of the samples, what would we expect to find?" The boxplots (Figure 3) indicate the medians / means / middle-50% / outliers among the answers to that question after asking it 100,000 times. A total of 10,927 unique students were involved in the sampling process: N(RiSE)=314 and N(Not-RiSE^A^)=10,613.

# Appendix F. DFW Rates in Gateway STEM courses

An important outcome variable for measuring student success in STEM is the failure rates in gateway courses. A common measure of this is the percentage of students in a course that received a D, F or W (withdrawal), called the DFW rate [48]. Students who receive a D, F or W do not meet the requirements for subsequent courses in a sequence or program, which can affect persistence. Previous literature indicated that students receiving a D, F, or W in introductory chemistry was strongly associated with changing to a non-STEM major [49,50]. The average DFW rates for the gateway mathematics and general chemistry courses for the duration of this study are shown in Table F1, as these are important to consider when interpreting course to course persistence patterns in math and chemistry.

**Table F1:** Average Rates of DFW (non-passing marks of D or F grades and Withdrawals) for mathematics and chemistry gateway courses at EC from Fall 2011 - Spring 2017.

| Course Name | Edmonds College Course Number | DFW Rate |
| --- | --- | --- |
| Intermediate Algebra (pre-college) | MATH 90 | 27% |
| Precalculus 1 | MATH& 141 | 29% |
| Precalculus 2 | MATH& 142 | 26% |
| Calculus 1 | MATH& 151 | 24% |
| Calculus 2 | MATH& 152 | 24% |
| Preparation for General Chemistry | CHEM 139 | 31% |
| General Chemistry 1 | CHEM& 161 | 19% |
| General Chemistry 2 | CHEM& 162 | 16% |

### 
